# Writing with AI boosts trust-building efficiency

**DOI:** 10.1016/j.isci.2025.114092

**Published:** 2025-11-20

**Authors:** Zoe A. Purcell, Maurice Jakesch, Mengchen Dong, Anne-Marie Nussberger, Nils Köbis

**Affiliations:** 1 LaPsyDÉ, Université Paris Cité, CNRS, 75005 Paris, France; 2 Bauhaus University, Weimar, Germany; 3 Center for Humans and Machines, Max Planck Institute for Human Development, Berlin, Germany; 4 Research Center Trustworthy Data Science and Security, University Duisburg-Essen, Duisburg, Germany

**Keywords:** Artificial intelligence, Emotion in artificial intelligence, Human-computer interaction, Social sciences, Psychology

## Abstract

AI-mediated communication promises efficiency but raises concerns about diminished authenticity and interpersonal trust. We examined this potential trade-off across two preregistered online experiments (*N* = 1,637) in which participants engaged in incentivized two-player trust games with communication. Half of the participants could use state-of-the-art predictive text assistance when composing a message to encourage trust; the others wrote unaided. We measured both objective (behavioral) and subjective (stated) trust. Frequentist and Bayesian analyses showed that AI assistance had minimal impact on trust, regardless of disclosure. AI-assisted participants wrote more efficiently, producing equally trust-inducing messages but in less time. This advantage persisted even when AI use was disclosed. Linguistic analyses indicated that AI-assisted messages were slightly less authentic than those written alone but that they exhibited greater warmth, complexity, and clout—features commonly associated with trustworthiness. These findings challenge the view that AI-mediated communication necessarily undermines trust, particularly in one-shot, transactional interactions.

## Highlights

• Those using AI-assisted writing gained similar levels of trust while investing less time
• Disclosure of AI use did not significantly lower trust levels
• Messages with AI help showed greater warmth, complexity, and clout
• Efficiency gains suggest that AI can support transactional trust-building

## Graphical abstract

**Figure:**
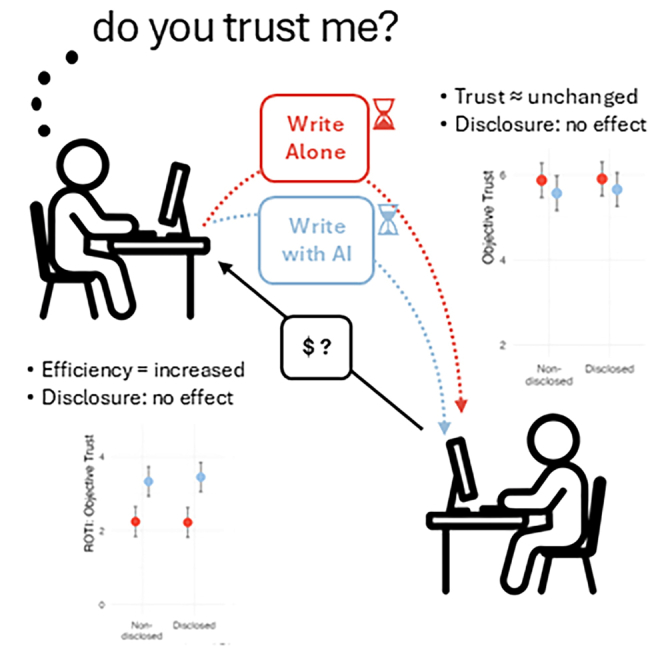

## Introduction

AI-mediated communication (AI-MC) is becoming integral to daily interactions, reshaping how we compose, communicate, and connect.^1^ Large language models (LLMs) such as ChatGPT, Gemini, and Claude now assist in drafting communications, from informal emails to official reports. One striking example is Meta’s integration of its LLaMA-based model into WhatsApp, embedding LLMs directly into everyday messaging and further normalizing their use in routine communication. However, as these technologies become more prevalent, recipients are increasingly alert to cues, such as overly polished language or specific phrases like “delve into,” that suggest AI, rather than the sender, may be shaping the communication.^2^^,^^3^ Beyond text generation, agentic AI tools such as Google’s Duplex or OpenAI’s Operator, which autonomously book reservations, have ignited debates over trust and transparency due to their highly human-like interactions.^4^ This wariness reflects a broader concern that while AI-MC promises efficiency and quality,^5^ it also threatens to disrupt trust and authenticity in communication.^6^ To explore these dynamics, we use trust-game paradigms in which participants compose messages with or without AI assistance, under varying conditions of transparency, and for real financial consequences contingent on trust.

Research highlights that efficiency in communication, especially in high-stakes or negotiated exchanges, is not merely about speed but about achieving desired relational or strategic outcomes with minimal cost or effort. In negotiation research, for instance, efficiency is defined as reaching satisfactory agreements while conserving time, effort, and relational capital.^7^ Organizational economics further emphasizes that trust itself can serve as a coordination mechanism, reducing the need for costly monitoring and enhancing both productivity and satisfaction, particularly when trust is mutual and internalized.^8^ We build on this broader framing by examining whether AI-assisted communication can increase such trust-building efficiency.

Trust is a complex and multifaceted phenomenon, fundamental to healthy and effective relationships.^9^^,^^10^ Like efficiency, definitions of trust vary widely by cultural, contextual, individual, and neural factors that are not necessarily mutually exclusive.^11^ However, popular accounts describe trust as operating along two principal routes: affective (emotion based) and cognitive (reason based).^12^^,^^13^ The relative dominance of each route depends on the nature of the interpersonal context. Affective trust is more likely to emerge in close, relational settings, whereas cognitive trust—the focus of the current work—plays a larger role in formal, transactional, or high-stakes interactions.

In empirical work, these routes are typically measured through three complementary lenses: individual (trait) trust, which captures judgments of a partner’s stable qualities (e.g., competence and integrity); subjective trust, which pertains to the trustor’s stated willingness to be vulnerable to a given partner in a specific interaction; and objective (behavioral) trust, which involves a costly act that places resources at risk.^14^ Our studies examine cognitive trust through individual, subjective, and objective lenses.

When considering trust and AI-MC, it is important to consider trust-related factors that may be specifically diminished or enhanced by AI use.^1^^,^^15^ Studies show that AI involvement in communication can shift perceptions of message ownership and accountability, affecting how communicators are judged.^16^ AI-generated language can introduce biases, such as excessive positivity, which may undermine perceptions of social attraction.^17^ AI-generated texts are also potentially perceived as less trustworthy than human-authored content.^6^^,^^18^ However, recent research also shows that AI-mediated communication can restore interpersonal trust in failed interactions by acting as a “moral crumple zone.”^19^ These mixed effects raise important questions about how AI involvement and its disclosure impact trust. For example, when AI-MC involvement is suspected or disclosed, and perceptions of agency shift, to what extent is the message still seen as an authentic expression of the human author’s intentions—and is it trusted accordingly?

A critical issue surrounding questions about efficiency, trust, and the future of AI-mediated communication is disclosure. Recent regulatory efforts, such as the EU AI Act, emphasize the importance of disclosing when and how AI is used, largely treating AI involvement as binary rather than graded. For AI systems classified as high risk, including human-interactive systems like LLM-based chatbots, transparency is mandatory, and the systems must disclose themselves.^20^ The policy situation becomes more complicated when AI merely plays an intermediary role and mediates human communication. Here, current disclosure mandates do not seem to apply. For instance, people do not have to disclose whether a written message was (co-)drafted with language models such as ChatGPT, leaving the disclosure of AI involvement to individual discretion. At the same time, recent research shows that undisclosed AI use in communication can be (ab)used for malicious purposes^21^^,^^22^ and that people find undisclosed AI use unacceptable.^23^

The current study responds to the unclear regulatory situation by providing initial experimental evidence of how AI-mediated communication and different transparency policies affect interpersonal, cognitive trust, and communication efficiency. Unlike previous studies that rely on hypothetical scenarios or stated preferences, we employ a machine behavior approach, examining real algorithms and actual human behavior in dynamic, incentivized interactions.^17^^,^^24^

**Figure 1:**
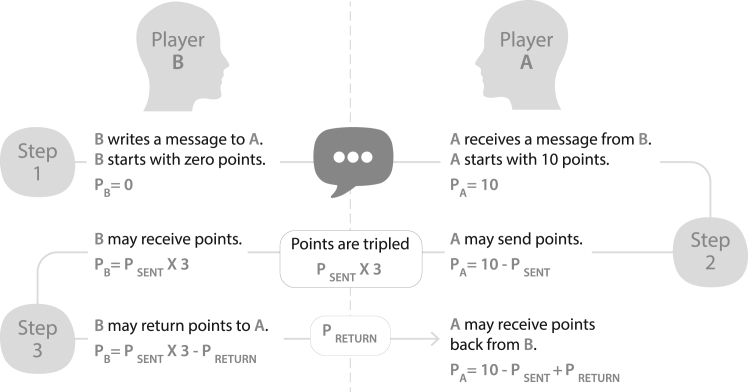
Adapted trust game setup In the adapted version of the trust game, player Bs write a message to player A to build trust and persuade them to send more points. P_A_, number of points held by player A. P_B_, number of points held by player B.

We use the well-established trust game—a canonical paradigm in behavioral economics and social psychology.^25^^,^^26^^,^^27^ In its classic form, the trust game captures the willingness to accept vulnerability, a core feature across disciplinary definitions of trust. Player A is endowed with a sum of money and chooses how much to send to player B, with the amount tripled. Player B then decides how much to return (see Figure 1). The game models many real-world trust situations, particularly those involving cognitive trust, where the interaction is more transactional, marked by interdependence and social uncertainty, allowing researchers to quantify trust and reciprocity in a controlled setting.

Building on this foundation, we implemented a modified version of the trust game that incorporates a communication phase prior to the transfer. In our version, player B was asked to send a short message to player A to elicit their trust.^28^^,^^29^ Sending such messages has been shown to influence trust dynamics by affecting perceived benevolence and competence.^30^ The more points player A sends to player B, the higher the trust. At the end of the game, each player earned 10 cents per point. In addition to this behavioral measure of objective trust, we also collected self-reported subjective trust (e.g., do you trust this person?) and perceived individual trustworthiness (e.g., is this person competent?).

All player Bs had to send a message to player As. In the treatment group, player Bs could use an AI writing assistant to compose their message to player As, while control group participants wrote the message without assistance. As with typical predictive text tools, this implies that players using the writing assistance tools could use none, some, or all the AI-generated suggestions and that they had the final say in what to send to their counterparts. We additionally compare non-disclosure, where AI use is unknown to the recipient, to scenarios where AI involvement is transparently communicated.

**Figure 2:**
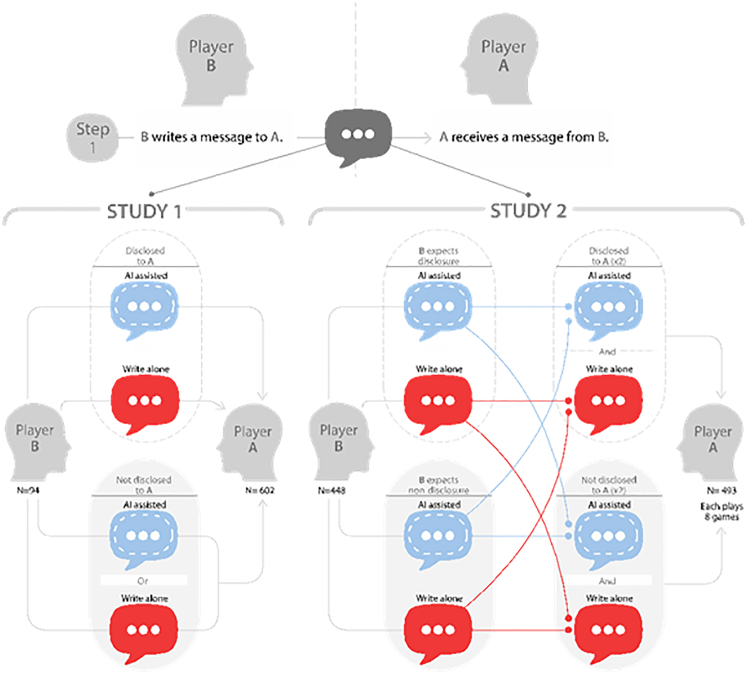
Experimental conditions in studies 1 and 2 In studies 1 and 2, player Bs wrote with or without AI. We also varied the actual and expected disclosure across studies.

We examine different variations of disclosure scenarios, shown in Figure 2, to test how disclosure affects trust. In study 1, player A was either shown whether player B had access to AI tools or not. At the same time, player B was unaware of the disclosure. In study 2, to examine how disclosure expectations mediate efficiency and trust, we introduced an additional treatment, where player B either expected their AI use to be disclosed to player A or not. Our findings contribute to the growing field of research exploring the impact of AI regulations in behavioral experiments,^31^ offering anticipatory, evidence-based insights to inform future policymaking and highlight the need for incorporating the impact of AI systems into existing theories of human-human trust.

The remainder of the paper is organized as follows: we first describe the experimental designs of studies 1 and 2, then introduce the outcome and efficiency measures, present the results for each study, and conclude with a joint discussion. A detailed description of the experimental and statistical methods is provided in the method details section.

**Figure 3:**
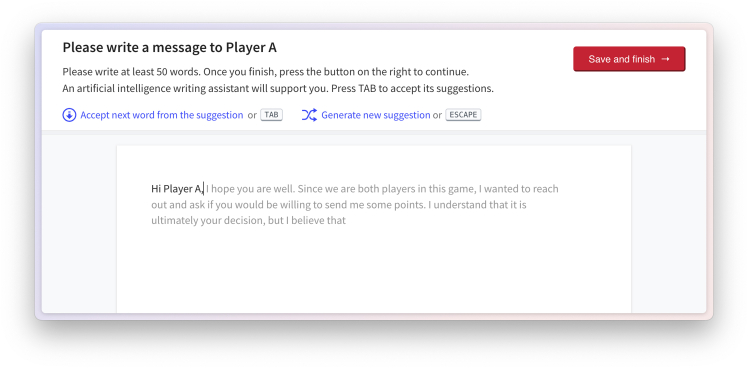
An example of the message instructions and predictive continuations for player B in the AI-assisted writing condition

As described in Figure 2, our manipulations of player B’s use of AI, expected disclosure, and actual disclosure occurred during step 1 of the adapted trust game. In study 1, player Bs were randomly assigned to write their message with (AI-assisted) or without AI (write-alone). See Figure 3 in STAR Methods for an example of the writing assistant. These messages were multiplied and sent to player As with or without disclosure. Player As in the AI-assisted + disclosed treatment received messages written with AI and labeled as such, and player As in the write-alone + disclosed treatment received messages written without AI and labeled as such. Player As in the non-disclosed treatment received a message written with or without AI but with no disclosure.

In study 2, as shown in Figure 2, player Bs’ expectations about disclosure were set, and each player A played eight games. Player As in the disclosed treatment saw all disclosed messages, while player As in the non-disclosed treatment saw all non-disclosed messages. Within each treatment and for each player A, expected disclosure and AI use were fully crossed. Hence, each player A saw four messages from player Bs who expected disclosure—two written with AI and two written without AI—and four messages from player Bs who did not expect disclosure—again, two written with AI and two written without AI. This design ensured full statistical control over player B’s AI use and expected and actual disclosure.

## Results

To assess how writing a trust-inducing message with AI and the disclosure of the AI use affect trust, we collected two central proxies for objective and subjective trust: we measured objective, behavioral trust as the points player A sent to player B, and subjective trust with player A’s self-reported ratings of how much they trust player B. We also considered individual trust via the three subcomponents, examining player A’s perceptions of player B’s competence, benevolence, and integrity.^14^ In addition to the experimental conditions, we used a continuous measure of AI involvement. This index was calculated by dividing the number of AI-generated words in each message by the total number of words in that message.

We also investigated how AI-assisted writing and disclosure impact communication efficiency. Insights for the conceptualization of communication efficiency stem from negotiation research, distinguishing between effectiveness (did trust occur?) and efficiency (how resourcefully it was achieved?).^7^ In line with that notion, we measure efficiency using well-established indices from economics, namely return on investment. Specifically, we calculated two return on investment indices (ROIIs): one based on the number of points sent (ROII_objective_) and another on subjective trust ratings (ROII_subjective_), both relative to the time spent writing. Exploratory linguistic analyses of AI-assisted and write-alone messages (pooled across studies 1 and 2) provide preliminary insights into qualitative differences between these conditions. For interested readers, supplementary analyses exploring the roles of age and gender on objective and subjective trust are provided in the supplemental information section 3. The analyses, unless otherwise stated as exploratory, were pre-registered prior to data collection (https://osf.io/nrwb8/).

### Study 1

To assess the impact of AI-assisted writing on cognitive trust, we compared three treatments under which player A received a single message from player B: (1) messages written without AI and disclosed as such (write-alone + disclosed group), (2) messages written with or without AI, without disclosure (non-disclosed treatment), or (3) messages written with AI and disclosed as such (AI-assisted + disclosed treatment). Successful manipulation checks are reported in the supplemental information 1.0. Data were analyzed using linear mixed models regressing treatment on our dependent variables with a random intercept effect for the respective message. We also conducted Bayesian analyses for our primary tests of the effect of treatment in the case of null results.

**Figure 4:**
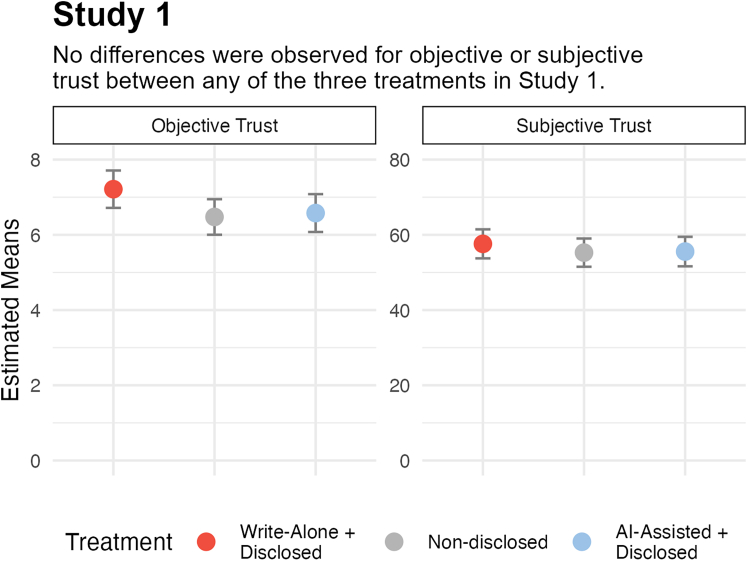
Study 1 results show the estimated means for objective (left) and subjective (right) trust by treatment Error bars reflect 95% confidence intervals.

As shown in Figure 4, there were no differences between treatments for objective (*F*(2, 294) = 2.72, *p* = 0.068, η^2^ = 0.02) or subjective trust (*F*(2, 273) = 0.44, *p* = 0.681, η^2^ <0 .01). These null effects were confirmed with Bayesian analyses: our objective trust data were 4× more likely under a model without treatment, and our subjective trust data were 37× more likely (for further details, see supplemental information 1.1). Similarly, we observe no differences between any of the three treatments for any of the individual trust components: competency, benevolence, or integrity (see supplemental information 1.2 for more details).

**Figure 5:**
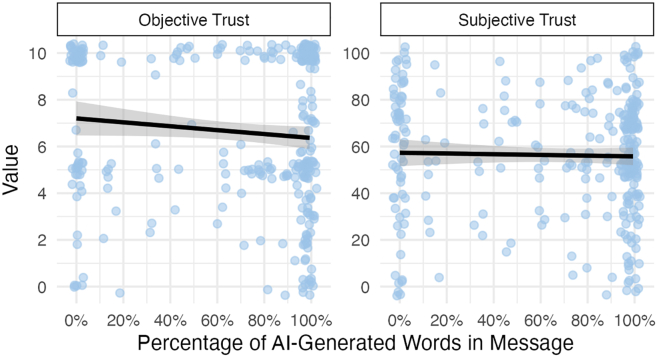
In study 1, for AI-assisted messages, a slight negative relationship was observed between the proportion of AI-generated words sent and the amount of objective trust garnered No relationship was observed between the proportion of AI-generated words and subjective trust. Shading reflects 95% confidence bands.

Exploratory analyses revealed that AI-assisted messages elicited greater objective trust when a lower proportion of the message was generated by AI (*F*(1, 48.9) = 3.25, *p* = 0.075, η^2^ = 0.06), but no such effect was observed for subjective trust (*F*(1, 46.5) = 0.20, *p* = 0.650, η^2^ < 0.01), as shown in Figure 5. However, the distribution of AI use was bimodal, with most participants generating either very little or nearly all of their message using AI. As such, inferences about moderate AI use should be treated with caution. That said, these results suggest that extensive reliance on AI-generated text may carry costs for behavioral trust, even if overall effects remain small.

Further exploratory testing showed that AI assistance also influenced ROIIs, corresponding to the observation that participants using AI writing tools had spent less time writing their messages, while eliciting similar levels of trust. In the non-disclosure treatment, player Bs, who wrote with AI, garnered higher objective (*F*(1, 76) = 4.77, *p* = 0.032, η^2^ = 0.06) and subjective trust (*F*(1, 78) = 6.70, *p* = 0.011, η^2^ = 0.06) per time spent composing messages. However, in the disclosed treatments, AI use did not affect the return on either objective (*F*(1, 82) = 1.06, *p* = 0.310, η^2^ = 0.01) or subjective trust (*F*(1, 80) = 1.98, *p* = 0.068, η^2^ = 0.02; see supplemental information 1.3 for more details). These ROII findings motivated the design of study 2, which systematically investigates the effect of different disclosure scenarios on trust and return on time invested.

### Study 2

In study 2, player As played a series of games reading multiple messages from different player Bs, some written with and some without AI assistance. At the same time, each player A consistently either experienced disclosure or non-disclosure across all messages (manipulation checks are reported in supplemental information 2.0). Data were analyzed using linear mixed models regressing AI use, expected disclosure, and actual disclosure on our dependent variables with random intercept effects for respective participants and messages.

**Figure 6:**
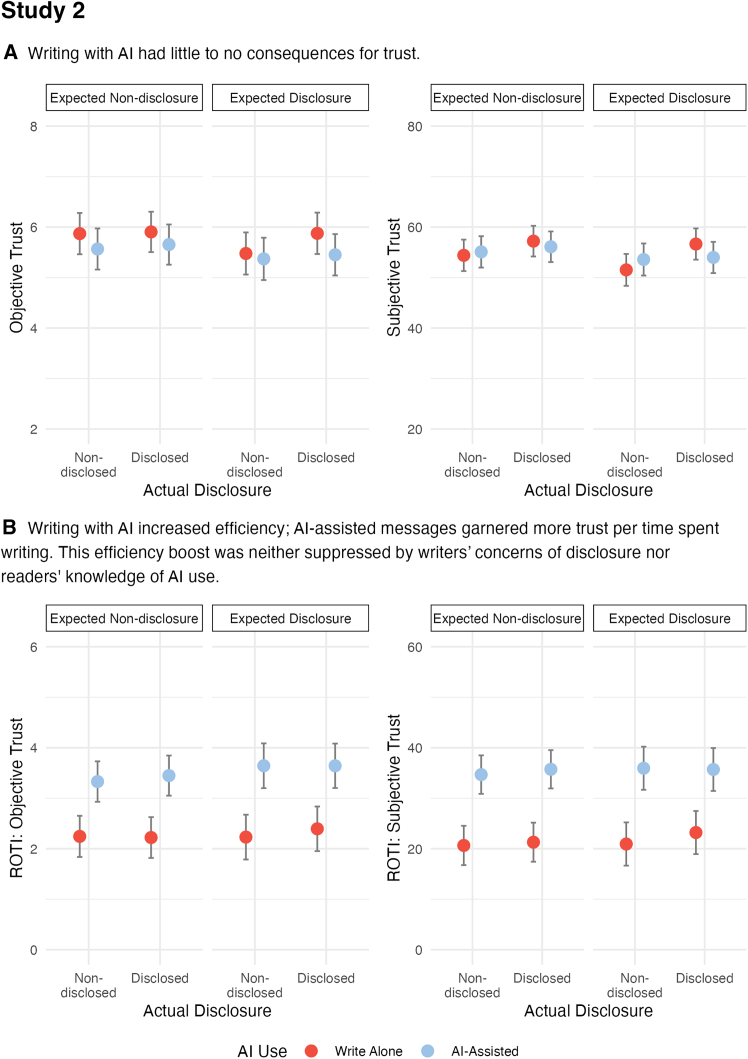
Study 2 results show the estimated means for objective and subjective trust (top) and objective and subjective ROIIs (bottom) Error bars reflect 95% confidence intervals.

As shown in Figure 6, AI use in study 2 had little to no effect on trust. Yet, we do observe large, positive effects of AI use on efficiency, replicating the findings of study 1. To unpack these findings a bit more, even with the increased sample and an immediate, heightened contrast between AI-assisted and write-alone messages through the experimental treatments, we observed only a small effect of AI use on objective trust (*F*(1, 394.68) = 5.21, *p* = 0.023, η^2^ = 0.01). That is, when averaged across actual and expected disclosure, messages written alone yielded only marginally more points from player A than those with AI assistance. No other relevant factors or interactions were observed for objective trust. Regarding subjective trust, AI use had no effect (*F*(1, 365.72) = 0.09, *p* = 0.761, η^2^ <0 .01), nor did any other factors or interactions in the model. Relatedly, neither AI use, expected disclosure, nor actual disclosure had meaningful effects on any of the three components of individual trust: competence, benevolence, or integrity (see supplemental information 2.2).

In contrast, AI use had a substantial and consistent positive effect on efficiency. Player Bs using AI assistance built more trust per minute of writing than those writing without AI, regardless of disclosure status. These efficiency increases were observed for both objective (*F*(1, 427.06) = 41.66, *p* <0 .001, η^2^ = 0.09) and subjective trust (*F*(1, 423.98) = 52.94, *p* <0 .001, η^2^ = 0.11). As shown in Figure 5, when averaged across actual and expected disclosure, ROII_objective_ and ROII_subjective_ were significantly higher for player Bs with AI assistance than those writing alone.

**Figure 7:**
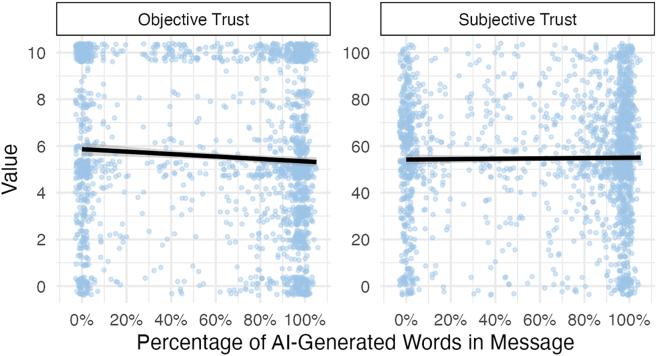
Similar to study 1, AI-assisted messages had a slight negative relationship between the proportion of AI-generated words sent and the amount of objective trust garnered No relationship was observed between the proportion of AI-generated words and subjective trust. Shading reflects 95% confidence bands.

As in study 1, exploratory analyses in study 2 showed that AI-assisted messages elicited greater objective trust when a smaller proportion of the message was generated by AI (*F*(1, 207.1) = 4.98, *p* = 0.027, η^2^ = 0.02), but this pattern did not hold for subjective trust (*F*(1, 183.5) = 0.31, *p* = 0.570, η^2^ < 0.01), as shown in Figure 7. However, as in study 1, the distribution of AI use was highly bimodal, with few participants exhibiting intermediate levels of usage. Inferences about the effects of moderate AI use should be interpreted with caution.

These results indicate that using AI to assist in writing may not undermine but facilitate trust building rather than detract from it. Furthermore, this efficiency boost persisted across expected and actual disclosure. That is, expected disclosure did not reduce the efficiency gain by writers taking extra time to avoid sounding “too AI-like.” In cases with disclosed AI use, readers did not suppress the efficiency boost by penalizing AI-assisted writers for using AI. For more details about ROII observations in study 2, see supplemental information 2.3.

### Linguistic analysis of messages

To explore how linguistic differences between AI-assisted and write-alone may have affected trust, we analyzed message content using Linguistic Inquiry and Word Count (LIWC).^32^ LIWC is a manually validated text analysis library widely used to quantify psychologically meaningful dimensions of word use, including those linked to trustworthiness and sincerity.^33^^,^^34^ Based on this research, we examined four LIWC cues: *analytical thinking* (structured, logical expression associated with competence),^35^ *clout* (confident, high-status language conveying authority),^36^ *authenticity* (honest, personal expression inversely linked to deception),^37^ and emotional *tone* (overall affective valence related to warmth and interpersonal trust).^38^ We also include the frequency of *numbers* and *personal pronouns*, along with three markers of effort and complexity: *message length*, *words per sentence*, and proportion of *big words* of more than six letters. These variables were included to explore whether elaborate or carefully crafted messages may be perceived as more trustworthy or thoughtful.^39^

**Figure 8:**
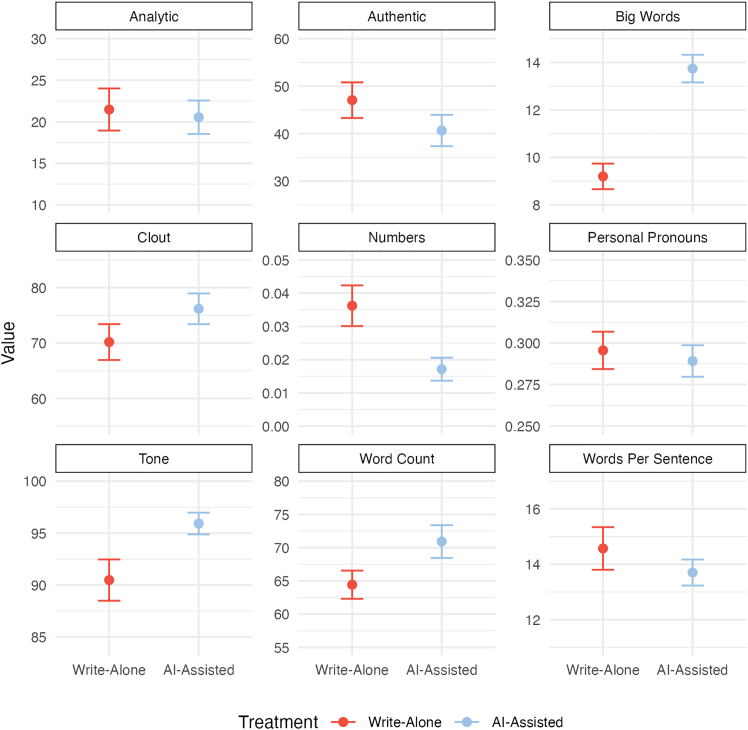
Across studies, messages written with AI assistance were more emotionally warm but less authentic, while being longer and including more complex words

As described in Figure 8, AI-assisted messages had lower authenticity scores, *t*(540) = −2.52, *p* = 0.012, and contained fewer number-related words than write-alone messages, *t*(540) = −5.40, *p* < 0.001. Additionally, AI-assisted messages contained more complex words, *t*(540) = 11.27, *p* < 0.001, and more words in total, *t*(540) = 3.91, *p* < 0.001. Similarly, clout, *t*(540) = 2.78, *p* = 0.006, and tone scores were significantly higher for AI-assisted messages, *t*(539) = 4.83, *p* < 0.001. There was a marginal trend toward fewer words per sentence in AI-assisted messages compared to the write-alone messages, *t*(540) = −1.92, *p* = 0.056. However, there was no difference between the write-alone and AI-assisted messages on analytic thinking scores, *t*(540) = −0.57, *p* = 0.570, or personal pronouns, *t*(540) = −0.86, *p* = 0.390.

## Discussion

Our findings, drawn from two incentivized trust game experiments, challenge the widespread belief that using AI writing tools may undermine cognitive trust in communication. For the most part, recipients (player As) were not less trusting of player Bs who had used AI and did not “punish” senders of messages written with AI assistance by returning smaller amounts in the trust game when AI usage was disclosed. In contrast, people writing with AI were more efficient at garnering trust, and this efficiency persisted when that use was transparent.

Our findings are aligned with previous behavioral research testing the effect of transparency of algorithmic presence.^31^ Specifically, in advice-giving contexts, disclosing (vs. not) that advice text was generated by AI did not significantly impact advice adoption by recipients. Possibly due to the growing exposure of LLMs in daily interactions and the belief that especially others are using such tools widely,^23^ people might have become accustomed to AI-mediated communication.

The finding that disclosure of AI use did not meaningfully reduce cognitive trust in our studies may reflect deeper psychological mechanisms of trust calibration at play. Namely, people adjust their trust based on expectations and context.^40^ When users already suspect AI involvement or see it as normative, disclosure may confirm rather than violate expectations.^41^ The distinction between expected and actual disclosure also matters. If disclosure aligns with what users anticipated, it is less likely to trigger distrust. In contrast, unexpected disclosures may feel deceptive. These dynamics suggest that disclosure effects are filtered through prior beliefs and evolving norms. It is important to note, however, that the current study examined only some forms of disclosure—a mandatory, system-imposed disclosure (in both studies 1 and 2) that was made explicit to player Bs in advance of message writing (in study 2). These design features may have shaped player Bs’ communication and behavior and influenced recipients’ reactions, depending on their (un)favorable attitudes toward AI-MC tools and the broader positive or negative framing of such tools. For instance, when recipients know that disclosed AI tools were provided mandatorily (vs. voluntarily), they may be more likely to view them as legitimate, which can mitigate trust erosion.^42^ Rather than rendering disclosure irrelevant, our results highlight the need to design it thoughtfully, in line with users’ expectations. Indeed, before policy mandates are considered, further work should systematically investigate both the expected signaling value and the actual trust effects of disclosure across varying conditions, including its timing, voluntariness, and contextual and normative approval.

Another explanation could be that *how* AI crafts trust-building messages is relevant. In our study, we only examine whether the involvement of AI affects trust. Our findings cannot speak to whether people might react differently when they receive more information about the type (e.g., drafting, editing, and correcting the message) or extent of involvement (e.g., how much of the message is AI written),^43^ nor about how the form, clarity, or content of the disclosure may impact user reactions. However, our findings facilitate future work in this direction by providing two preliminary, exploratory analyses.

Our first exploratory investigation showed that a continuous measure of AI involvement was associated with small negative effects on objective, but not subjective, trust. However, interpretation should be tempered due to the bimodal distribution of AI usage. The second exploratory investigation yielded clearer findings. A linguistic analysis revealed notable differences: AI-assisted messages were more linguistically complex, contained fewer number-related terms, and exhibited a warmer tone and higher perceived clout. These features counter common stereotypes of AI as cold and mechanical, suggesting that current LLMs are capable of producing cues typically associated with trustworthiness. At the same time, AI-assisted messages were rated as less authentic. While this effect was in the expected direction, it was relatively small. In the context of other trust-promoting cues and continued algorithmic improvements, perceived authenticity may become a less central determinant of AI-MC trust than previously assumed. Future research should examine whether disclosing the extent of AI involvement impacts trust systematically and explore whether these linguistic features generalize to other AI-writing tools.

Although using AI to draft the message did not increase absolute, cognitive trust, it was still more efficient for senders to rely on the AI writing assistant. Namely, they received more points per time spent writing. Hence, AI-mediated communication tools can increase efficiency when establishing human-human trust. Efficiency, however, is not purely transactional. Prior work has shown that expectations, shared understanding, and trust climate critically shape how efficiently communication unfolds.^7^ Our finding that disclosure did not undermine efficiency suggests that transparent AI use may foster a trust climate that supports streamlined coordination. However, whether this advantage will extend to higher-stakes environments or other relations, for example, between romantic partners, patient and doctor, or politician and constituent, is unclear. Relatedly, while our studies suggest that *cognitive* trust is not affected, it may be that *affective* trust is impacted, especially in more personal or higher-stakes environments. Future studies should investigate how AI assistance impacts the trust-efficiency trade-off in these more specific contexts and with measures of affective trust.

A rich body of literature has developed around human-human trust, often emphasizing interpersonal factors such as cognition, affect, and situation awareness as critical in trust formation and maintenance.^12^^,^^44^^,^^45^ However, these traditional frameworks focus on direct human interactions, neglecting how increasingly agentive AI systems can mediate trust between humans. Our results indicate that AI-mediated communication, through efficient trust elicitation, can play an essential role in trust-building. We see a fruitful path forward in integrating AI into human trust frameworks as a mediating force that can streamline trust dynamics in human interactions.

Our findings also carry implications for emerging transparency regulations. While disclosure did not harm cognitive trust or efficiency in our setting, such outcomes are likely context dependent and shaped by user expectations. As trust calibration is sensitive to norm shifts and interaction history,^40^ disclosure policies should be designed to align with users’ mental models and not assume one-size-fits-all effects. We therefore caution against premature regulatory conclusions and advocate for further interdisciplinary research to develop adaptive and context-sensitive AI disclosure guidelines.

While AI tools like writing assistants can increase the speed and ease of communication, this emphasis on efficiency raises broader societal concerns about the potential effects of AI-mediated communication on the richness of human relationships. Currently, AI seems to facilitate cognitive trust-building in brief interactions. However, there is a risk that over-reliance on AI for everyday communication could lead to shifting norms and long-term effects such as a decline in critical thinking and/or social skills due to ongoing, regular delegation of these tasks to machines. Hence, while immediate AI-MC use may boost trust-building efficiency, there may be long-term and societal-level consequences.

### Limitations of the study

While this study provides valuable insights into AI’s role in communication and trust, several limitations must be noted. First, although the trust game offers strong internal validity and is widely used in behavioral research, it remains a highly abstract, emotionally neutral interaction. As such, its ecological validity is limited, and our findings may not generalize to emotionally rich or high-stakes contexts where trust is relational, dynamic, and culturally nuanced (e.g., professional collaborations, healthcare interactions, or political discourse). Nevertheless, this abstraction allows for tight experimental control, making it a useful starting point for identifying core mechanisms before extending to more complex real-world scenarios—particularly for studying cognitive trust and one-shot, transactional interactions. Future work should aim to test these findings in more naturalistic and emotionally charged environments.

Second, our study focused on a general form of AI involvement in message creation using predictive text assistance, without differentiating between types or degrees of AI mediation (e.g., editing, suggesting, or fully generating text). Although this choice reflects real-world predictive systems, it limits our ability to identify which aspects of AI involvement are most consequential for trust formation. Our exploratory linguistic analysis offers some insight into the qualitative characteristics of AI-assisted messages, but more in-depth analysis is needed to understand how message tone, complexity, and style contribute to observed trust effects.

Third, while we observed gains in trust-building efficiency—as measured by the amount of trust elicited per unit of time spent composing messages—this metric focuses on a specific behavioral outcome and does not address other important relational factors (cf. Butler and Chami and Fullenkamp^7^^,^^8^). Faster communication is not necessarily better in all contexts nor for all relational factors. In many domains, trust is built through deliberation, emotional resonance, and shared meaning. Thus, efficiency gains observed here should be considered as context dependent. The ethical implications of this speed-trust trade-off also warrant attention: in domains such as healthcare, education, or governance, prioritizing speed may undermine important factors like empathy, reflection, and accountability, making such gains not only ineffective but also potentially harmful (see Dang and Liu, Purcell and Bonnefon, and Kee and Knox^45^^,^^46^^,^^47^). Future work should therefore examine how efficiency interacts with communicative depth and other critical relational factors across different contexts.

Fourth, our study involved only one-shot interactions, which do not reflect the iterative and cumulative nature of trust in ongoing relationships (for a discussion, see Alós-Ferrer and Farolfi^11^). Long-term or relational trust—where individuals build shared histories and expectations—may be influenced differently by AI mediation. We did not assess whether the trust gains observed are stable over time, justified by message content, or potentially lead to misplaced trust or overreliance. These concerns are particularly important in high-stakes domains like healthcare, education, and governance, where persistent interaction and accountability are critical. It is possible, for instance, that while AI-assisted communication accelerates cognitive trust in initial encounters, repeated use could either strengthen trust—by supporting fast, consistent, and clear exchanges—or undermine it if overreliance on AI, for example, diminishes affective trust or accountability. In affect-rich relationships, efficiency may matter less than qualities such as empathy and shared understanding, meaning our observed benefits might attenuate, reverse, or take on new forms when trust develops across multiple interactions. Longitudinal studies will be essential to examine how AI use affects the development, maintenance, and repair of trust over time.

Finally, both studies used US-based online participants, limiting the cultural scope of our findings. Since cultural norms significantly shape perceptions of trust and AI, cross-cultural replications will be important to assess the generalizability of our conclusions.^48^^,^^49^ Differences in expectations of transparency, emotional tone, and trustworthiness could all influence the effects of AI-mediated communication in global settings. Our samples also did not allow systematic analysis of individual factors such as AI familiarity or digital literacy, which may moderate how people interpret and respond to AI-assisted messages. Taken together, our findings provide an initial benchmark for US participants, but future research should broaden the cultural and demographic base and consider individual experience with AI to refine these insights.

### Conclusions

Our findings indicate that AI-assisted communication can foster cognitive trust efficiently and effectively, suggesting a valuable role for AI in certain types of interpersonal exchanges. However, as AI becomes more embedded in social interactions, it is crucial to remain vigilant about its potential long-term impacts on relationship quality and social cohesion.^50^ Presently, however, the technologically driven efficiency gains do not appear to come at the cost of cognitive trust, nor are they jeopardized by imposing disclosure, particularly in the case of one-shot, transactional interactions.

## Resource availability

### Lead contact

Further information and requests for resources should be directed to and will be fulfilled by the lead contact, Zoe A. Purcell (purcell.z.a@gmail.com).

### Materials availability

This study did not generate new materials.

### Data and code availability

• Study designs and analyses were preregistered at https://osf.io/nrwb8/, except where noted as exploratory.
• Anonymized data have been deposited at OSF and are publicly available at https://osf.io/nrwb8/.
• Analysis code is available at https://osf.io/nrwb8/.

## Acknowledgments

This work was supported by the National French Research Agency (ANR-23-AERC-0006) and by the Research Center Trustworthy Data Science and Security (RC Trust).

## Author contributions

Z.A.P. and M.J. had full access to all of the data in the study and took responsibility for the integrity of the data and the accuracy of the data analysis. All authors reviewed the manuscript and agreed to be accountable for all aspects of the work. Conceptualization, M.J., Z.A.P., A.-M.N., M.D., and N.K.; protocol implementation and data collection, Z.A.P.; software development, M.J.; data analysis, M.J. and Z.A.P.; writing – original draft, Z.A.P.; writing – review and editing, M.J., Z.A.P., A.-M.N., M.D., and N.K.; visualization, Z.A.P.; funding acquisition, Z.A.P. and N.K.

## Declaration of interests

The authors declare no competing interests.

## STAR★Methods

### Key resources table

**Table:** 

REAGENT or RESOURCE	SOURCE	IDENTIFIER
**Deposited data**		

Raw and analyzed data	This paper	https://osf.io/nrwb8

### Experimental models and study participant details

Study 1 included 602 Player As (representative of U.S. demographics) and 94 Player Bs (non-representative), with an overall age range of 18–94 years (*M* = 44.58, *SD* = 15.77) and 52% women. Study 2 had 493 Player As and 448 Player Bs (both representative of U.S. demographics), with an overall sample of 971 participants aged 18 to 83 years (*M* = 46.01, *SD* = 15.70) and 50% women. Comprehension questions were presented at the outset of the study; participants who gave incorrect responses twice could not continue through the study.

Our sample sizes were determined based on prior studies examining similar effects.^26^^,^^27^ Additionally, our use of a diverse and representative sample (where applicable) enhances the generalizability of our findings. All studies were granted full ethical approval by the Max Planck Institute for Human Development, Berlin, Germany [AI-mediated trust; A 2023–01]. Informed consent was obtained from all participants, and no deception was used.

### Method details

Study designs and analyses were preregistered at OSF (https://osf.io/nrwb8/), except where noted as exploratory. In both studies, Player Bs composed a single message for Player A – half with the assistance of an AI writing tool (AI-assisted) and half independently (write-alone). The AI tool, similar to Google’s Smart Compose and Microsoft’s Copilot, provided text suggestions as participants wrote. The suggestions were shown inline and could be simply written over or accepted by pressing TAB. Suggestions were generated automatically whenever participants paused their writing for about 1.5 s and could be actively requested with the escape key. The backend for these suggestions was an adapted GPT-3.5 model, primed with context on the game and instructions. Figure 3 contains an example of the interface for Player Bs in the AI-assisted condition.

#### Study 1

Study 1 explored how specific combinations of AI assistance and the disclosure of its use influenced trust from message recipients, Player As. Participants included an online U.S. sample aged 18 to 94 (*M* = 44.58, *SD* = 15.77), with 52% women. Player Bs (*N* = 94, non-representative sample) received a base payment of $2, and Player As (*N* = 602, representative of U.S. age, ethnicity, and gender demographics) received a base payment of $1. All games were incentivized, with each point earned converting to a 10-cent bonus.

We employed a strategy method in two phases. In phase 1, Player Bs were randomly assigned to either the AI-assisted or write-alone condition. Each Player B composed a message for Player A and indicated how many points they would return for each possible amount Player B might receive (ranging from 0 to 30). In phase 2, Player As were randomly assigned to one of three conditions: non-disclosed, AI-assisted + disclosed, or write-alone + disclosed. In the non-disclosed condition, Player As were informed that Player B might have used an AI tool but were not told whether AI was actually used. In the AI-assisted disclosed condition, Player As were informed that Player B could use an AI tool to co-draft the message. In the write-alone disclosed condition, Player As were informed that Player B could not use AI.

Each Player A read a single message and played one game. After reading the message, Player As chose how many points to transfer (0–10) and rated their subjective trust in Player B (0 = Do not trust at all, 100 = Completely trust). They also rated Player B’s perceived benevolence, competence, and integrity, indicated whether they thought AI was used, and to what extent. Messages from Player Bs were reused across games, allowing statistical control over message content and writer.

#### Study 2

Study 2 was designed to investigate cross-player effects and the potential advantages of AI assistance for writers. Additionally, it examined whether expectations about AI disclosure or actual disclosure affected efficiency. To that end, we increased the sample of Player Bs and set expectations about disclosure, informing them whether Player A would be told about their AI use. The sample included an online U.S. cohort (*N* = 971) aged 18 to 83 (*M* = 46.01, *SD* = 15.70), with 50% women. Both Player Bs (*N* = 448, representative) and Player As (*N* = 493, representative) received a base payment of $2. At least one game per participant was incentivized, with each point earned translating to a 10-cent bonus. Paid games always aligned expected disclosure with actual disclosure to ensure no deception was introduced.

Using a strategy method again, in phase 1, Player Bs were randomly assigned to one of four conditions: AI-assisted + expected non-disclosure, AI-assisted + expected disclosure, write-alone + expected non-disclosure, or write-alone + expected disclosure. In phase 2, Player As participated in a mixed-design setup, playing a series of eight trust games. They read eight messages in total, each varying by Player B’s AI assistance (manipulated within subjects), expected disclosure (whether Player B anticipated disclosure or non-disclosure of AI use; manipulated within subjects), and actual disclosure (manipulated between subjects). As in Study 1, for each game, Player As decided how many points to send Player B, their level of trust in Player B, and responded to several additional questions.

### Quantification and statistical analysis

The results were analyzed primarily using linear mixed models, with random intercepts for messages and participants as appropriate. Bayesian analyses supplemented null findings for preregistered hypotheses. Effect sizes were reported using η^2^ for ANOVA-derived tests and supplemented by Bayes factors where relevant. No corrections for multiple comparisons were applied. All primary analyses were preregistered (https://osf.io/nrwb8/), and exploratory analyses—clearly indicated as such—included assessments of AI involvement as a continuous predictor, return on investment indices (ROIIs) for Study 1, and linguistic features derived from LIWC.
