# Supplementary material for: Writing with AI boosts trust-building efficiency
Source: iScience. 2025 Nov 20;28(12):114092. doi: 10.1016/j.isci.2025.114092 (PMC12765382; doi:10.1016/j.isci.2025.114092)
Supplement: Document S1. Figures S1–S4, Tables S1–S24, and supplementary material [file mmc1.pdf]

iScience, Volume *28*

Supplemental information

Writing with AI boosts trust-building efficiency

Zoe A. Purcell, Maurice Jakesch, Mengchen Dong, Anne-Marie Nussberger, and Nils Köbis

Supplementary Material

1. Study 1

To examine how treatment impacted trust, we ran a linear mixed model regressing a fixed effect of treatment and a random effect of message on objective trust (points sent from Player A to Player B), subjective trust, and how many points Player A expected Player B to return.

1.0 Manipulation Check

Table S1. The effect of treatment on beliefs that AI was used in Study 1.

DV	Deviance	Num. df	Den. df	p
Treatment	121.34	2	599	0

Table S2. Pairwise comparisons between treatments of beliefs that AI was used in Study 1.

Contrast	Estimate	SE	Z Ratio	p
Write-Alone + Disclosed - Non-Disclosed	-1.57	0.21	-7.37	0.000
AI-Assisted + Disclosed. - Non-Disclosed	-2.36	0.24	-9.67	0.000
Write-Alone + Disclosed - AI-Assisted + Disclosed	-0.79	0.24	-3.26	0.003

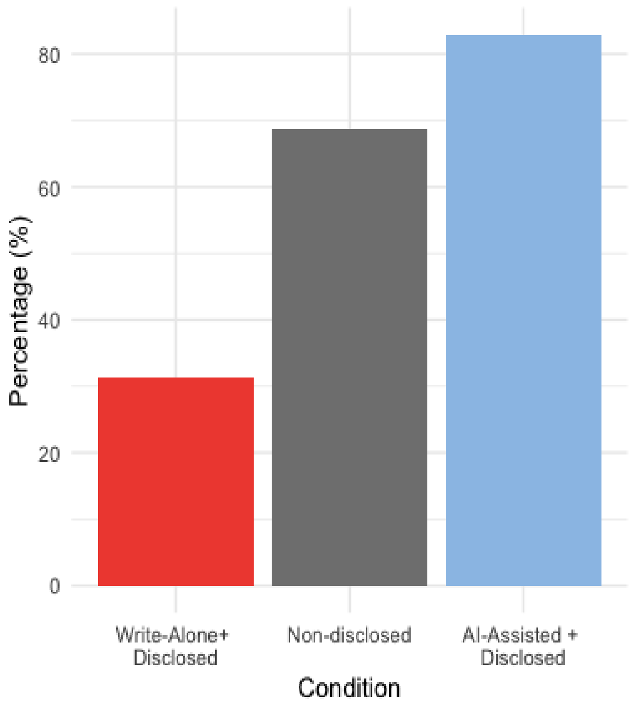

Figure S1. *The percentage of Player As who believed AI was used differed significantly between treatments, suggesting the manipulation was successful.*

1.1 Impact of Treatment on Trust

Table S3. ANOVA Results for objective trust, subjective trust, and expected returns in Study 1.

DV	F	Num. df	Den. df	Eta Sq.	p
Objective Trust	2.72	2	294.20	0.02	0.068
Subjective Trust	0.44	2	273.66	<.01	0.647
Expected Return	0.39	2	277.50	<.01	0.675

To quantify the evidence for the null effects of treatment, we calculated Bayes factors comparing the models with treatment to models without treatment. We used the *BayesFactor* library[1] with wide Cauchy distributions and the BFlinearModel approach with Jeffreys-Zellner-Siow priors.

Table S4. Bayesian ANOVA Results for objective Trust, subjective trust, and expected returns in Study 1.

DV	Bayes Factor	Our data is:
Objective Trust	0.26	3.9x more likely under the model without treatment.
Subjective Trust	0.03	36.8x more likely under the model without treatment.

DV	Bayes Factor	Our data is:
Expected Return	0.03	35.8x more likely under the model without treatment.

1.2 Impact of Treatment on Three Components of Trust: Competence, Benevolence and Integrity

Table S5. ANOVA Results for competence, benevolence, and integrity in Study 1. Note: No random effect for message was included for benevolence or integrity due to singularity – eta squared are reported instead of partical eta squared.

DV	F	Num. df	Den. df	Eta Sq.	p
Competence	2.50	2	238.64	0.02	0.084
Benevolence	0.91	2	599.00	<.01	0.403
Integrity	0.98	2	599.00	<.01	0.376

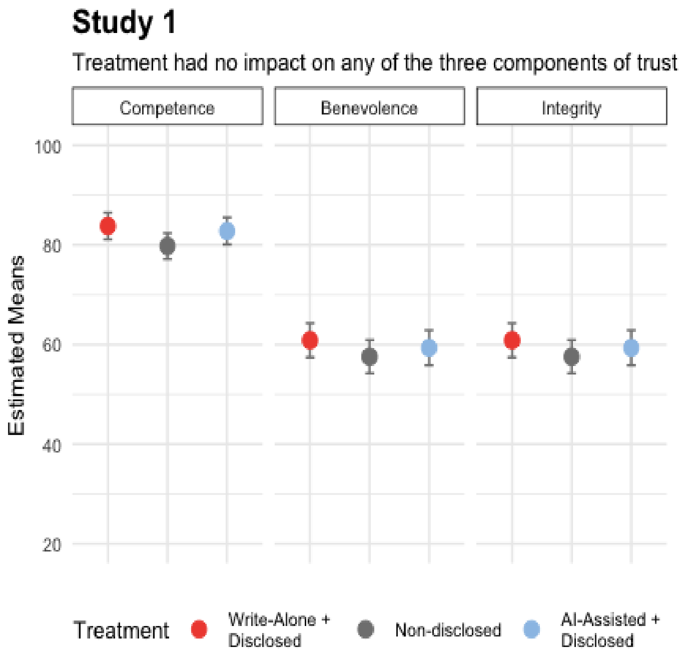

Figure S2. *Three components of trust: Estimated means for competence, benevolence, and integrity ratings of Player Bs. Error bars reflect 95% percent confidence intervals.*

1.3 Independent Effects of AI Use and Disclosure

In addition to our primary analyses above, we explored whether AI use, actual disclosure, and their interaction affected objective and subjective trust, and Return on Investment Indices (ROIIs).

1.3.1 Impact of AI Use and Disclosure on Trust

To explore how AI use and actual disclosure impacted trust, we ran linear mixed models regressing fixed effects of AI use, actual disclosure, and their interaction, and a random intercept effect of message on trust. For objective trust, the interaction of AI use and disclosure showed a marginal effect but no other effects were significant. For subjective trust, there were no significant effects. Moreover, examining the effects of AI use separately for non-disclosed and disclosed messages revealed no significant differences.

Table S6. ANOVA results for objective trust by AI use and actual disclosure in Study 1.

Effect	F	Den. df	Eta Sq.	p
AI Use	0.02	96.45	<.01	0.897
Actual Disclosure	2.18	594.93	<.01	0.140
AI Use * Actual Disclosure	3.83	594.93	0.01	0.051

Note: Numerator df for all effects in this model is 1.

Table S7. ANOVA results for subjective trust by AI use and actual disclosure in Study 1.

Effect	F	Den. df	Eta Sq.	p
AI Use	0.31	93.89	<.01	0.579
Actual Disclosure	0.30	594.99	<.01	0.585
AI Use * Actual Disclosure	2.02	594.99	<.01	0.156

Note: Numerator df for all effects in this model is 1.

Table S8. Exploratory ANOVA results showing the impact of AI use on trust within disclosed and non-disclosed conditions, respectively.

DV	F	Den. df	Eta Sq.	p
Objective trust: Disclosed	1.90	79.64	0.02	0.171
Subjective trust: Disclosed	0.44	73.46	0.01	0.511
Objective trust: non-disclosed	1.20	59.81	0.02	0.278
Subjective trust: non-disclosed	1.44	58.19	0.02	0.235

Note: Numerator df for all effects in this model is 1.

1.3.2 Impact of AI Use and Disclosure on Return on Time Invested Indices (ROIIs)

To explore how AI use and actual disclosure impacted ROIIs, we ran linear mixed models, as above, on ROIIs. For both ROII: objective trust and ROII: subjective trust, no effects in the full models were significant. However, when non-disclosed and disclosed messages were examined separately, ROIIs: objective and subjective were higher for messages written with AI than messages written alone.

Table S9. Overall ANOVA results showing the impact of the interaction between AI use and disclosure on ROII:Objective

Effect	F	Den. df	Eta Sq.	p
AI Use	2.33	91.46	0.02	0.131
Actual Disclosure	1.34	523.71	<.01	0.248
AI Use * Actual Disclosure	1.06	523.71	<.01	0.304

Note: Numerator df for all effects in this model is 1.

Table S10. Overall ANOVA results showing the impact of the interaction between AI use and disclosure on ROII:Subjective

Effect	F	Den. df	Eta Sq.	p
AI Use	3.38	90.34	0.04	0.069
Actual Disclosure	2.09	526.05	<.01	0.149
AI Use * Actual Disclosure	0.65	526.05	<.01	0.419

Note: Numerator df for all effects in this model is 1.

Table S11. Exploratory ANOVA results showing the impact of AI use within disclosed and non-disclosed conditions, respectively.

DV	F	Den. df	Eta Sq.	p
Objective trust: Disclosed	1.06	82.15	0.01	0.307
Subjective trust: Disclosed	1.98	82.15	0.02	0.163
Objective trust: non-disclosed	4.77	82.15	0.06	0.032
Subjective trust: non-disclosed	6.70	82.15	0.08	0.011

Note: Numerator df for all effects in this model is 1.

Overall, the results in Study 1 suggest that there was little or no effect of treatment on trust. However, exploratory findings show that there may be nuanced effects of AI use and actual disclose on trust and ROIIs.

2. Study 2

To optimize for exploring cross-player efficiency effects (ROIIs), we significantly increased both the number of Player Bs and the total number of games played. Additionally, we considered whether any observed efficiency gains may be moderated by Player B expectations about disclosure; that is, if informed that their AI use would be disclosed, Player Bs may invest more time in composition, jeopardizing any efficiency pay-off.

2.0 Manipulation Check

Table S12. The effect of treatment on beliefs that AI was used in Study 2.

Effect	Deviance	Residual df	Residual Deviance	p
Null		3,934	5,434.41	
AI Use	618.11	3,933	4,816.30	0
Actual Disclosure	15.36	3,932	4,800.94	0
AI Use * Actual Disclosure	289.29	3,931	4,511.65	0

Table S13. Pairwise comparisons between treatments of beliefs that AI was used in Study 2.

Contrast	Estimate	SE	Z Ratio	p
AI-Assisted Non-disclosed - Write-alone Non-disclosed	0.55	0.09	5.87	0
AI-Assisted Non-disclosed - AI-Assisted Disclosed	-1.44	0.10	-13.96	0
AI-Assisted Non-disclosed - Write-alone Disclosed	1.55	0.10	14.76	0
Write-alone Non-disclosed - AI-Assisted Disclosed	-1.98	0.10	-19.01	0
Write-alone Non-disclosed - Write-alone Disclosed	1.00	0.11	9.43	0
AI-Assisted Disclosed - Write-alone Disclosed	2.99	0.11	25.97	0

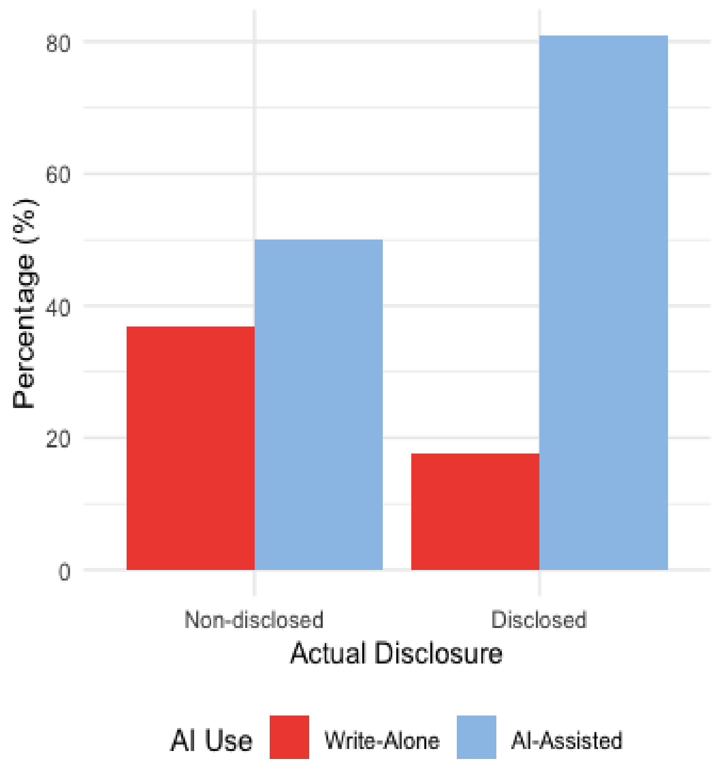

Figure S3. *The percentage of Player As who believed AI was used differed significantly between AI Use and Actual Disclosure conditions, suggesting the manipulation was successful.*

2.1 Impact of AI Use, Expected- and Actual Disclosure on Trust

To examine the effects of AI use, actual- and expected disclosure on trust, we used linear mixed models with fixed effects for effects of AI use, actual- and expected disclosure, and random intercepts for participant and message. AI use had a small but significant effect on objective trust. Follow-ups revealed that 'write alone' messages (*eM*=5.78, *SE*=0.14) garnered more objective trust than 'AI assisted' messages (*eM*=5.51, *SE*=0.14*).

Table S14. Overall ANOVA results showing the impact of AI use, actual- and expected disclosure on objective trust.

Effect	F	Den. df	Eta Sq.	p
AI Use	5.21	394.68	0.01	0.023
Actual Disclosure	0.43	491.23	<.01	0.514
Expected Disclosure	2.96	394.64	0.01	0.086
AI Use * Actual Disclosure	0.82	3,293.07	<.01	0.367
AI Use * Expected Disclosure	0.00	394.68	<.01	0.961
Actual * Expected Disclosure	1.51	3,298.33	<.01	0.219

Effect	F	Den. df	Eta Sq.	p
AI Use * Actual Disclosure * Expected Disclosure	1.63	3,298.47	<.01	0.202

Note: Numerator df for all effects in this model is 1.

The model for subjective trust revealed a significant main effect of expected disclosure and interaction effect of AI use and actual disclosure. Follow-ups revealed that subjective trust was higher for messages without expected disclosure (*eM*=55.7, *SE*=1.02) than with disclosure expected (*eM*=53.93, *SE*=1.04). Additionally, follow-ups of the interaction effect of AI use by actual disclosure revealed that, critically, there was no difference between write alone and AI assisted messages for either non-disclosed conditions (*t*=1.29, *p*=0.196) or disclosed conditions (*t*=-1.81, *p*=0.071). Rather, this interaction stemmed from a difference between non-disclosed (*eM*=52.96, *SE*=1.41) and disclosed messages (*eM*=56.93, *SE*=1.38) that were written alone (*t*=-2.11, *p*=0.035) compared to when written with AI assistance (*t*=-0.38, *p*=0.706).

Table S15. Overall ANOVA results showing the impact of AI use, actual- and expected disclosure on subjective trust.

Effect	F	Den. df	Eta Sq.	p
AI Use	0.09	365.72	<.01	0.765
Actual Disclosure	1.73	491.43	<.01	0.189
Expected Disclosure	4.31	365.67	0.01	0.039
AI Use * Actual Disclosure	7.02	3,355.79	<.01	0.008
AI Use * Expected Disclosure	0.00	365.75	<.01	0.961
Actual * Expected Disclosure	0.48	3,359.60	<.01	0.490
AI Use * Actual Disclosure * Expected Disclosure	1.40	3,359.69	<.01	0.236

Note: Numerator df for all effects in this model is 1.

2.2 Impact of AI use, expected- and actual disclosure on the three components of trust.

Table S16. ANOVA results showing the impact of AI use, actual- and expected disclosure on competence ratings.

Effect	F	Den. df	Eta Sq.	p
AI Use	0.72	383.33	<.01	0.396
Actual Disclosure	0.21	491.46	<.01	0.645

Effect	F	Den. df	Eta Sq.	p
Expected Disclosure	2.12	383.29	0.01	0.146
AI Use * Actual Disclosure	0.82	3,306.67	<.01	0.365
AI Use * Expected Disclosure	0.10	383.34	<.01	0.751
Actual * Expected Disclosure	1.00	3,311.77	<.01	0.317
AI Use * Actual Disclosure * Expected Disclosure	1.89	3,311.89	<.01	0.169

Table S17. ANOVA results showing the impact of AI use, actual- and expected disclosure on benevolence ratings. Despite a significant interaction between AI use and actual disclosure–no pairwise comparisons were significant after adjustments for multiple comparisons.

Effect	F	Den. df	Eta Sq.	p
AI Use	0.00	369.97	<.01	0.951
Actual Disclosure	2.77	491.11	0.01	0.097
Expected Disclosure	2.31	369.92	0.01	0.130
AI Use * Actual Disclosure	4.64	3,356.58	<.01	0.031
AI Use * Expected Disclosure	0.69	370.00	<.01	0.406
Actual * Expected Disclosure	0.77	3,360.39	<.01	0.380
AI Use * Actual Disclosure * Expected Disclosure	0.26	3,360.48	<.01	0.608

Table S18. ANOVA results showing the impact of AI use, actual- and expected disclosure on integrity ratings. As above, despite a significant interaction between AI use and actual disclosure–no pairwise comparisons were significant after adjustments for multiple comparisons.

Effect	F	Den. df	Eta Sq.	p
AI Use	0.30	363.00	<.01	0.583
Actual Disclosure	2.63	491.62	0.01	0.106
Expected Disclosure	1.57	362.95	<.01	0.212
AI Use * Actual Disclosure	7.29	3,371.16	<.01	0.007
AI Use * Expected Disclosure	2.48	363.03	0.01	0.116
Actual * Expected Disclosure	0.44	3,374.54	<.01	0.508

Effect	F	Den. df	Eta Sq.	p
AI Use * Actual Disclosure * Expected Disclosure	0.76	3,374.60	<.01	0.385

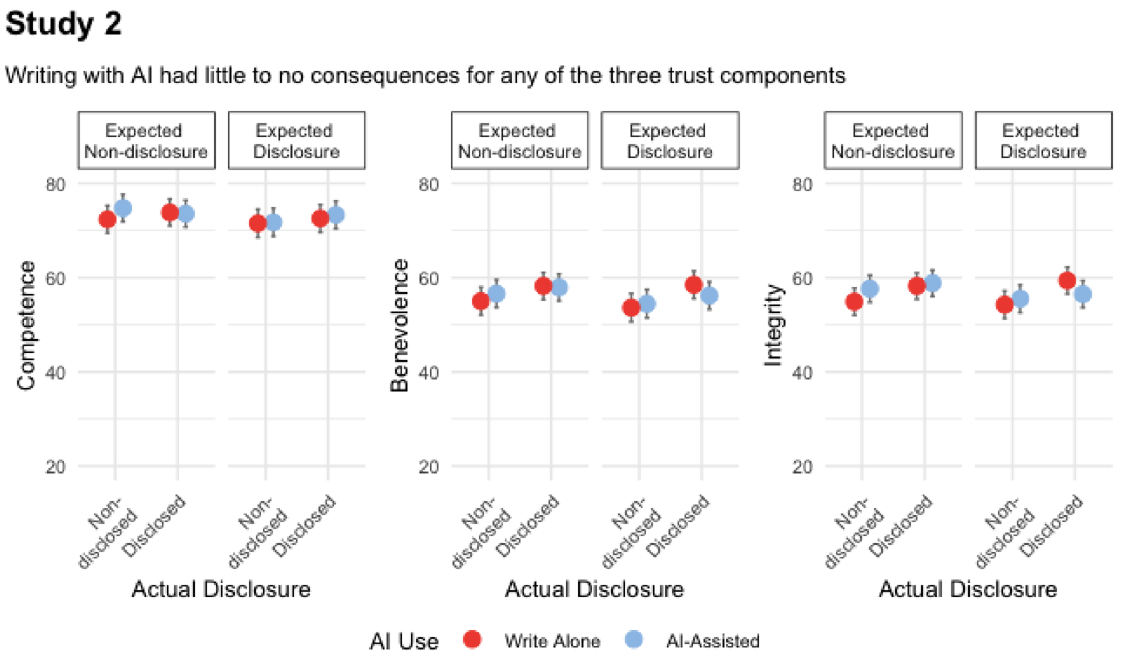

Figure S4. *Three components of trust: Estimated means for competence, benevolence, and integrity ratings of Player Bs. Error bars reflect 95% percent confidence intervals.*

2.3 Impact of AI Use, Expected- and Actual Disclosure on ROIIs

To examine the effects of AI use, actual- and expected disclosure on ROIIs, we used linear mixed models with fixed effects for effects of AI use, actual- and expected disclosure, and random intercepts for participant and message. Averaged across expected and actual disclosure, AI use had a large effect on ROII: objective trust. Follow-ups revealed that ROII: objective was higher for 'AI assisted' messages (*eM*=3.52, *SE*=0.15) than 'write alone' messages (*eM*=2.27, *SE*=0.15).

Table S19. Overall ANOVA results showing the impact of AI use, actual- and expected disclosure on ROII: Objective.

Effect	F	Den. df	Eta Sq.	p
AI Use	41.66	427.06	0.09	0.000
Actual Disclosure	0.27	490.93	<.01	0.604
Expected Disclosure	0.75	427.10	<.01	0.388
AI Use * Actual Disclosure	0.01	3,083.57	<.01	0.913

Effect	F	Den. df	Eta Sq.	p
AI Use * Expected Disclosure	0.20	427.07	<.01	0.651
Actual * Expected Disclosure	0.10	3,089.60	<.01	0.752
AI Use * Actual Disclosure * Expected Disclosure	2.04	3,089.38	<.01	0.153

The model for ROII: subjective trust revealed a large effect of AI use. Averaged across expected and actual disclosure, AI use had a large effect on ROII: subjective trust. As for ROII:objective, follow-ups revealed that ROII:subjective was higher for 'AI assisted' messages (*eM*=35.51, *SE*=1.42) than 'write alone' messages (*eM*=21.52, *SE*=1.44).

Table S20. Overall ANOVA results showing the impact of AI use, actual- and expected disclosure on ROII: Subjective

Effect	F	Den. df	Eta Sq.	p
AI Use	52.95	423.98	0.11	0.000
Actual Disclosure	0.92	491.92	<.01	0.339
Expected Disclosure	0.20	424.02	<.01	0.656
AI Use * Actual Disclosure	1.38	3,062.76	<.01	0.239
AI Use * Expected Disclosure	0.02	423.99	<.01	0.899
Actual * Expected Disclosure	0.03	3,068.86	<.01	0.855
AI Use * Actual Disclosure * Expected Disclosure	2.64	3,068.60	<.01	0.104

Note: Numerator df for all effects in this model is 1.

3. Demographics, Treatment, and Trust

3.1 Study 1

To examine how treatment, age and gender impacted trust, we ran a linear mixed model regressing a fixed effect of treatment, age and gender, and all possible interactions with a random effect of message on objective trust (points sent from Player A to Player B), subjective trust.

Table S21. ANOVA Results for objective trust with demographics in Study 1.

Effect	F	Den. df	Eta Sq.	p
Condition	2.71	348.61	0.02	0.068
Age	0.74	577.60	<.01	0.391

Effect	F	Den. df	Eta Sq.	p
Gender	0.43	571.64	<.01	0.512
Condition * Age	0.00	571.80	<.01	0.997
Condition * Gender	2.45	567.83	0.01	0.087
Age * Gender	1.34	567.78	<.01	0.248
Condition * Age * Gender	0.40	567.30	<.01	0.672

Table S22. ANOVA Results for subjective trust with demographics in Study 1.

Effect	F	Den. df	Eta Sq.	p
Condition	0.46	335.07	<.01	0.630
Age	8.68	582.81	0.01	0.003
Gender	1.48	580.29	<.01	0.224
Condition * Age	2.05	579.35	0.01	0.130
Condition * Gender	0.84	576.69	<.01	0.433
Age * Gender	3.53	577.08	0.01	0.061
Condition * Age * Gender	0.77	577.16	<.01	0.463

3.2 Study 2

To examine the effects of AI use and demographics on trust, we used linear mixed models with fixed effects for effects of AI use, expected- and actual-disclosure, and age and gender, and all their interactions with random intercepts for participant and message.

Table S23. ANOVA Results for objective trust with demographics in Study 2.

Effects	F	Den. df	Eta Sq.	p
AI Use	6.03	396.95	0.01	0.015
Expected	0.39	478.12	<.01	0.531
Actual	2.98	396.90	0.01	0.085
Age	2.74	477.84	0.01	0.099
Gender	0.04	477.49	<.01	0.836
AI Use * Expected	0.34	3,231.73	<.01	0.558

Effects	F	Den. df	Eta Sq.	p
AI Use * Actual	0.00	396.90	<.01	0.980
AI Use * Age	1.83	3,236.75	<.01	0.176
AI Use * Gender	14.51	3,230.70	<.01	0.000
Expected * Actual	0.15	477.73	<.01	0.702
Expected * Age	5.16	3,227.43	<.01	0.023
Expected * Gender	3.26	3,210.59	<.01	0.071
Actual * Age	3.05	477.73	0.01	0.082
Actual * Gender	5.16	3,213.69	<.01	0.023
Age * Gender	0.12	478.14	<.01	0.726
AI Use * Expected * Actual	1.67	3,236.73	<.01	0.196
AI Use * Expected * Age	4.01	3,227.62	<.01	0.045
AI Use * Expected * Gender	0.17	3,230.21	<.01	0.676
AI Use * Actual * Age	1.79	3,228.44	<.01	0.181
AI Use * Actual * Gender	6.28	3,224.61	<.01	0.012
AI Use * Age * Gender	0.30	3,212.07	<.01	0.587
Expected * Actual * Age	1.51	3,225.66	<.01	0.220
Expected * Actual * Gender	0.29	3,232.15	<.01	0.593
Expected * Age * Gender	0.89	478.06	<.01	0.347
Actual * Age * Gender	3.17	3,229.16	<.01	0.075
AI Use * Expected * Actual * Age	1.71	3,231.11	<.01	0.191
AI Use * Expected * Actual * Gender	0.99	3,221.34	<.01	0.321
AI Use * Expected * Age * Gender	4.67	3,235.19	<.01	0.031
AI Use * Actual * Age * Gender	1.00	3,232.24	<.01	0.319
Expected * Actual * Age * Gender	0.09	3,235.33	<.01	0.762
AI Use * Expected * Actual * Age * Gender	1.98	3,241.94	<.01	0.160

Considering multiple tests, most of the effects in the model were relatively small with the exception of the AI Use * Gender interaction. To follow up the significant interaction between AI Use and Gender, we examined the simple effects of AI Use (AI-assisted vs. write-alone) separately for male and female participants. For female participants, there was no significant difference in points sent between AI and Write-alone conditions, t(729) = -1.13, p = .26, 95% CI [-0.43, 0.11], estimate = -0.16, SE = 0.14. In contrast, male participants sent significantly fewer points in the AI assisted condition compared to the Write-alone condition, t(687) = -3.07, p = .0023, 95% CI [−0.69, −0.15], estimate = -0.42, SE = 0.14. These effects were estimated while averaging over levels of actual and expected disclosure, and degrees of freedom were calculated using the Kenward-Roger method. Given these effects are quite small and these analyses were not preregistered, these findings should be considered tentative until apriori confirmation is achieved.

Table S24. ANOVA Results for stated trust with demographics in Study 2.

Effects	F	Den. df	Eta Sq.	p
AI Use	0.26	367.05	<.01	0.614
Expected	1.85	478.41	<.01	0.174
Actual	4.21	367.02	0.01	0.041
Age	5.41	478.13	0.01	0.020
Gender	1.28	477.86	<.01	0.258
AI Use * Expected	4.91	3,292.04	<.01	0.027
AI Use * Actual	0.02	367.03	<.01	0.879
AI Use * Age	0.80	3,295.56	<.01	0.370
AI Use * Gender	11.45	3,289.55	<.01	0.001
Expected * Actual	0.21	478.04	<.01	0.647
Expected * Age	6.56	3,287.49	<.01	0.010
Expected * Gender	0.56	3,271.44	<.01	0.453
Actual * Age	1.08	478.08	<.01	0.300
Actual * Gender	1.37	3,273.58	<.01	0.242
Age * Gender	0.04	478.47	<.01	0.851
AI Use * Expected * Actual	1.79	3,295.66	<.01	0.181
AI Use * Expected * Age	3.57	3,286.72	<.01	0.059
AI Use * Expected * Gender	0.94	3,289.09	<.01	0.333

Effects	F	Den. df	Eta Sq.	p
AI Use * Actual * Age	1.91	3,286.83	<.01	0.167
AI Use * Actual * Gender	5.61	3,284.63	<.01	0.018
AI Use * Age * Gender	0.22	3,272.40	<.01	0.643
Expected * Actual * Age	0.20	3,285.46	<.01	0.657
Expected * Actual * Gender	5.75	3,295.07	<.01	0.017
Expected * Age * Gender	0.46	478.33	<.01	0.497
Actual * Age * Gender	1.12	3,292.70	<.01	0.290
AI Use * Expected * Actual * Age	2.45	3,289.19	<.01	0.118
AI Use * Expected * Actual * Gender	0.01	3,282.19	<.01	0.919
AI Use * Expected * Age * Gender	0.05	3,294.65	<.01	0.827
AI Use * Actual * Age * Gender	0.84	3,295.07	<.01	0.358
Expected * Actual * Age * Gender	0.01	3,295.12	<.01	0.905
AI Use * Expected * Actual * Age * Gender	4.14	3,299.93	<.01	0.042

As above, most of the effects were relatively small with the exception of the AI Use * Gender interaction. To probe this interaction for subjective trust we conducted simple effects analyses separately by gender. For female participants, there was no significant effect of AI use on trust, $t(729) = -1.13$, $p = .260$, 95% CI [−0.43, 0.11], estimate = -0.16, SE = 0.14. In contrast, for male participants, those receiving AI assisted messages trusted the sender less than those with Write-alone messages, $t(687) = -3.07$, $p = .002$, 95% CI [−0.69, −0.15], estimate = -0.42, SE = 0.14. These estimates are adjusted for the levels of actual and expected disclosure, with degrees of freedom computed using the Kenward-Roger approximation. For the same reasons stated above, these findings should be considered tentative until they are confirmed with further studies.

References

1. Morey R, Rouder J (2023). *BayesFactor: Computation of Bayes Factors for Common Designs*. R package version 0.9.12-4.6, https://github.com/richarddmorey/bayesfactor.
